# Transcriptomic responses to emamectin benzoate in Pacific and Atlantic Canada salmon lice *Lepeophtheirus salmonis* with differing levels of drug resistance

**DOI:** 10.1111/eva.12237

**Published:** 2014-12-22

**Authors:** Ben J G Sutherland, Jordan D Poley, Okechukwu O Igboeli, Johanna R Jantzen, Mark D Fast, Ben F Koop, Simon R M Jones

**Affiliations:** 1 Centre for Biomedical Research, Department of Biology, University of VictoriaVictoria, BC, Canada; 2 Institut de Biologie Intégrative et des Systèmes (IBIS), Département de biologie, Université LavalQuébec, QC, Canada; 3 Hoplite Lab, Department of Pathology and Microbiology, Atlantic Veterinary College, University of Prince Edward IslandCharlottetown, PEI, Canada; 4 Pacific Biological StationNanaimo, BC, Canada

**Keywords:** drug resistance, emamectin benzoate, polygenic resistance, salmon aquaculture, sea lice, transcriptomics

## Abstract

Salmon lice *Lepeophtheirus salmonis* are an ecologically and economically important parasite of wild and farmed salmon. In Scotland, Norway, and Eastern Canada, *L. salmonis* have developed resistance to emamectin benzoate (EMB), one of the few parasiticides available for salmon lice. Drug resistance mechanisms can be complex, potentially differing among populations and involving multiple genes with additive effects (i.e., polygenic resistance). Indicators of resistance development may enable early detection and countermeasures to avoid the spread of resistance. Here, we collect sensitive Pacific *L. salmonis* and sensitive and resistant Atlantic *L. salmonis* from salmon farms, propagate in laboratory (F1), expose to EMB in bioassays, and evaluate either baseline (Atlantic only) or induced transcriptomic differences between populations. In all populations, induced responses were minor and a cellular stress response was not identified. Pacific lice did not upregulate any genes in response to EMB, but downregulated degradative enzymes and transport proteins at 50 ppb EMB. Baseline differences between sensitive and now resistant Atlantic lice were much greater than responses to exposures. All resistant lice overexpressed degradative enzymes, and resistant males, the most resistant group, overexpressed collagenases to the greatest extent. These results indicate an accumulation of baseline expression differences related to resistance.

## Introduction

Development of parasiticide resistance in endo- and ectoparasites of importance to veterinary or human health is a major issue globally. Chemical control is ubiquitous across many parasite taxa, with administration ease and initial efficacy leading to over-reliance, and in some cases resistance (Sangster 2001). For example, trichostrongylid nematode parasites of sheep are commonly resistant to the major classes of anthelmintics, which can limit sheep production (Gilleard 2006). Although currently much less severe than that which occurs in nematodes of sheep, resistance to anthelmintics in nematodes of horses and cattle suggests a similar future concern (Kaplan and Vidyashankar 2012). Control of the cattle tick *Rhipicephalus microplus* is limited by the development of resistance to the acaricides used to treat this and other tick species (Abbas et al. 2014). Alongside agriculturally important parasites, mosquito vectors of malaria are becoming resistant to chemical treatments throughout Africa (Ranson et al. 2011), and the head louse *Pediculus humanus capitis* is also becoming resistant to commonly used parasiticides in North America, leading to increased infestations (Yoon et al. 2014). New drugs are slow and expensive to develop (Kaplan and Vidyashankar 2012), and thus, the preservation of efficacy of existing treatments is critical (Sangster 2001; Kaplan 2004) through various management and husbandry strategies and reduction of reliance on chemical control (Kaplan 2004; Kaplan and Vidyashankar 2012).

A major challenge to salmon aquaculture is the recent development of resistance of salmon lice to several important chemical control methods. Salmon lice are ectoparasitic copepods that feed on skin, mucus, and blood of wild and farmed salmon causing host damage and potentially facilitating secondary infections (Fast 2014). Parasiticide treatments are used to remove lice from farmed salmon. As a result, the aquaculture industry is largely reliant on chemical control (Johnson et al. 2004; Igboeli et al. 2014) with limited diversity of mechanisms of action, increasing the potential for resistance development (Denholm et al. 2002). A commonly used in-feed treatment is emamectin benzoate (EMB; trade name SLICE™, Merck), a macrocyclic lactone avermectin derivative that is thought to bind parasite glutamate-gated chloride channels causing hyperpolarization in neuromuscular cells, paralysis, and death (Arena et al. 1995; Stone et al. 1999; Glendinning et al. 2011). Medicated feed is given over a 7-day period and EMB accumulates in flesh and mucus (at 70–100 ppb when administered at 50 μg/kg) (Sevatdal et al. 2005). Resistance to EMB has emerged in *Lepeophtheirus salmonis* in Norway (Espedal et al. 2013), Scotland, and Atlantic Canada (Lees et al. 2008; Jones et al. 2012a, 2013; Carmichael et al. 2013) as well as in *Caligus rogercresseyi* in Chile (Bravo et al. 2008), whereas lice in Western Canada remain sensitive (Saksida et al. 2013). This is an important issue for the health and welfare of wild and domestic salmon.

Pesticide resistance can occur by direct disruption of target site binding, metabolism, and detoxification of the active product, increased compound efflux, or reduced uptake through the cuticle or digestive lining (i.e., penetration resistance) (Clark et al. 1995; Bonizzoni et al. 2012; Igboeli et al. 2012). Metabolic resistance is typically polygenic, with many small and additive effects, whereas target site disruption can be monogenic, with a large effect (i.e., knockdown resistance) (ffrench-Constant et al. 2004). Resistance mechanisms can also occur together. For example, complementary mechanisms may explain geographic variation in dichlorodiphenyltrichloroethane (DDT) resistance in knockdown-resistant (*kdr*) mosquitoes (Brooke 2008; Donnelly et al. 2009). Importantly, selective pressures leading to monogenic or polygenic resistance typically differ. Polygenic resistance is favoured when drug exposures occur within a range of tolerance for a portion of the population, whereas monogenic resistance is favoured when exposure occurs outside the range of tolerance (see ffrench-Constant et al. 2004). Genomics and transcriptomics enable exploration of complementary resistance mechanisms, such as metabolism or sequestration (ffrench-Constant et al. 2004; David et al. 2005), and can also be useful in monitoring and managing resistance (Pedra et al. 2004; Vontas et al. 2005; Zhao et al. 2006).

In Atlantic and Pacific *L. salmonis* subspecies (Yazawa et al. 2008; Skern-Mauritzen et al. 2014), the dynamics, mechanisms, or potential of EMB resistance are active areas of study. Resistant *L. salmonis* populations were collected from salmon farms in Norway and propagated for four generations, or crossed with a sensitive laboratory strain (Espedal et al. 2013). Pure strains remained resistant through the generations, hybrids were of intermediate sensitivity between resistant and sensitive strains, and no reproductive or survival costs were associated with resistance. Also indicating a lack of costs, Atlantic Canada *L. salmonis* remained highly resistant over three generations without continued selection (Igboeli et al. 2013). Intermediate hybrid sensitivity suggests polygenic resistance (Espedal et al. 2013) as identified in other species to ivermectin (for review see Clark et al. 1995). In some cases, resistance may be due to increased efflux of EMB by P-glycoprotein (pgp), for example, through upregulation of *p-glycoprotein* transcription in response to EMB presence (Heumann et al. 2012) or increased baseline levels of *p-glycoprotein* mRNA (Igboeli et al. 2012). Male-specific *p-glycoprotein* upregulation in response to EMB has also been identified, with resistant lice increasing expression to a greater extent than sensitive (Igboeli et al. 2013). Baseline and induced (at 200 ppb EMB) transcriptome differences were recently explored in male *L. salmonis* from Scottish populations with differing EMB sensitivity; the largest effect identified was between populations regardless of EMB presence, and the authors highlighted reduced expression of a *GABA-gated chloride channel* and a *neuronal acetylcholine receptor* in the resistant population in response to EMB, among others (Carmichael et al. 2013). Whether EMB resistance in *L. salmonis* involves multiple factors and the specifics of these factors are yet to be determined.

Here, we apply a 38K oligonucleotide microarray (Sutherland et al. 2012; Yasuike et al. 2012) and reverse-transcription quantitative PCR (RT-qPCR) to profile gene expression responses of sensitive Pacific lice to EMB (0, 10, 25, 50 ppb) and to compare baseline and induced (0, 0.1, 25, 300, 1000 ppb) expression differences between sensitive and resistant Atlantic lice (Igboeli et al. 2013). Sex-specific differences in EMB resistance have been identified (Westcott et al. 2008; Igboeli et al. 2013; Jones et al. 2013), and accordingly, we profile transcriptomic responses in both male and female pre-adult Atlantic *L. salmonis*. Our study provides the first baseline dataset for EMB transcriptome responses of the Pacific subspecies of *L. salmonis* and provides new insight into resistance mechanisms in the Atlantic subspecies.

## Materials and methods

### Pacific salmon lice collection, EMB exposure, and RNA extraction

Pacific *L. salmonis* were obtained from Atlantic salmon *Salmo salar* farms near Campbell River, British Columbia (BC), in March 2009. From these individuals, nauplius larvae were hatched and grown to copepodids, which were allowed to attach to laboratory-reared Atlantic salmon as previously described (Sutherland et al. 2014). After 40 days, salmon were sedated with metomidate hydrochloride (Aquacalm, Syndel Laboratories Ltd.) and pre-adult lice were collected (777 individuals). Stock emamectin benzoate (PESTANAL®, Sigma–Aldrich, St. Louis, MO, USA) was prepared to 100 mg/L in methanol and then diluted to working concentrations in seawater. After removal from fish, lice were held briefly in 10°C seawater for <1 h. Approximately 25 pre-adult stage I and II male and female individuals were haphazardly distributed into each of 24 beakers containing 500 ml filtered and aerated seawater (10°C; 30 parts per thousand (ppt) salinity). Groups of six beakers were assigned to four EMB concentrations (0, 10, 25, or 50 ppb; Table1), and temperature was maintained at 10°C by incubation in a water bath. After 24 h, lice from each beaker were collected on a mesh filter and flash-frozen (*n* = 24 pools). Total RNA was extracted from frozen tissue using TRIzol® (Life Technologies, Carlsbad, CA, USA) followed by RNeasy column purification (Qiagen, Venlo, Netherlands), as per manufacturers' instructions. Purified total RNA was tested by agarose gel electrophoresis for quality and by spectrophotometry (NanoDrop-1000) for purity and quantity. Use of research animals complied with Fisheries and Oceans Canada Pacific Region Animal Care Committee protocol number 09-001.

**Table 1:** Experimental design for RNA profiling. Layout of experiment displaying the number of biological replicates for each condition. Biological replicates are individuals for Atlantic and pools of ∽25 lice for Pacific lice

Subspecies	Stage	Sex	Emamectin benzoate (EMB) resistance	Sample size for each [EMB concentration (ppb)]				
				[0]	[10]	[25]	[50]	
Pacific	Pre-adult	Mixed	Sensitive	6	6*	6	6	
				[0]	[0.1]	[25]	[300]	[1000]
Atlantic	Pre-adult	Female	Low	4	4	4	4	4
			High	4	4	4	2	4
		Male	Low	4	4	3	4	4
			High	4	4	4	4	4

* Condition used for RT-qPCR only.

### Atlantic salmon lice collection, EMB exposure, and RNA extraction

Atlantic *L. salmonis* were collected in 2012 from Atlantic salmon farms in Back Bay (resistant) and near Grand Manan, New Brunswick (sensitive) (Jones et al. 2012a). Live lice were moved to the Atlantic Veterinary College (University of Prince Edward Island) and grown on Atlantic salmon for approximately 80–90 days until the extrusion of the third set of egg strings. Larvae from these egg strings were grown to copepodids and then allowed to rear to the pre-adult stage on Atlantic salmon (Covello et al. 2012; Igboeli et al. 2013). Subsequently, salmon were sedated with tricaine methanesulfonate (MS-222), and lice were collected in Petri dishes with seawater (10°C, 33 ppt salinity). Petri dishes were swirled and only adherent lice were used in subsequent studies. EMB exposures were performed as per standardized bioassay protocols (Westcott et al. 2008). There have been no observable differences in gene expression, phenotype, or survival from the concentration of methanol (EMB solvent) in the EMB dilutions (Hoplite laboratory *personal communication*), and standardized bioassay protocols do not call for a solvent control (Westcott et al. 2008). Therefore, here, we did not include both a seawater and solvent control. Lice from each population were separated by sex, and four individuals were distributed to each of 40 flasks containing seawater (10°C, 33 ppt salinity). The flasks were assigned in duplicate to 20 treatment groups: EMB concentration (0, 0.1, 25, 300, 1000 ppb), sex, and population (resistant, sensitive; Table1). EMB concentrations were selected to include those used in Pacific lice exposures (e.g., 25 ppb) and those known to cause phenotypic effects in resistant Atlantic lice (e.g., 1000 ppb; M. Fast, *personal observation*). After 24 h, lice from each beaker were flash-frozen individually and kept at −80°C until RNA extraction (Table1). Total RNA was extracted as described above, with the exception of using TURBO DNase treatment (Life Technologies) prior to RNeasy column purification (Qiagen). Purified total RNA was tested by agarose gel and automated electrophoresis (Experion; Bio-Rad, Hercules, CA, USA). Use of research animals for this study was approved by Canadian Animal Care Committee protocol #12-016.

To confirm EMB sensitivity differences between the resistant and sensitive populations, 24-h bioassays were performed on a subset of the parental collection (F0) and on lice propagated in laboratory (F1). For F0 lice, these bioassays were performed in duplicate flasks with 10 lice per flask per condition (i.e., for each sex, population, and concentration combination), and for F1 lice, with five and four lice per condition for resistant and sensitive populations, respectively (F1 EC50 bioassay sample sizes were constrained to reserve lice for RNA profiling bioassays). After 24 h, lice were evaluated as per standards (Westcott et al. 2008) and EC50 values were calculated in GraphPad (v6; Prism, La Jolla, CA, USA).

### cDNA preparation and microarray hybridization

For each sample, 825 ng of total RNA was amplified to Cy5-labeled cRNA using the Low-Input Quick Amp system (Agilent; v6.5, Santa Clara, CA, USA). A Cy3-cRNA reference pool used to hybridize alongside samples (Churchill 2002) was generated for the Pacific and Atlantic lice experiments, ensuring the inclusion of at least one individual from all experimental conditions. The sample and reference pool combination was hybridized to oligonucleotide microarrays designed using previously annotated ESTs from both Pacific and Atlantic *L. salmonis* (Yasuike et al. 2012; eArray design ID 024389; Agilent), scanned on a ScanArray Express (Perkin Elmer, Waltham, MA, USA) and quantified on Imagene (v8.1; BioDiscovery, Hawthorne, CA, USA) as previously reported (Sutherland et al. 2012). For each probe, the background median was subtracted from the foreground, samples were imported into GeneSpring GX11 (Agilent), any negative raw values were changed to 1.0, and each array was normalized by intensity-dependent *Lowess* normalization (Yang et al. 2002) (Agilent).

### Transcriptome analyses

Pacific and Atlantic experiments were analyzed separately. For each experiment, quality control filters retained probes passing the following criteria in at least 65% of the samples in any one condition: no poor quality flags and raw signal ≥ 500 in both channels.

Pacific lice exposures to 0, 25, and 50 ppb (*n* = 6 pools per condition; Table1) were profiled by microarray (the 10 ppb condition was only included in subsequent RT-qPCR analysis). Differential expression was tested by one-way anova without equal variance assumption (Benjamini–Hochberg multiple test corrected *P* ≤ 0.01), followed by *post hoc* Tukey HSD (*P* ≤ 0.01) and fold change filter (FC ≥ 1.5) between conditions.

Atlantic male and female pre-adult lice from sensitive and resistant populations exposed to 0, 0.1, 25, 300, and 1000 ppb were profiled by microarray (*n* = 20 conditions; Table1). Differential expression was tested by three-way anova (Benjamini–Hochberg multiple test corrected *P* ≤ 0.01) using population, sex, and EMB concentration as factors and including interaction effects. Each condition contained four biological replicates except for two conditions that had insufficient high-quality RNA (i.e., male sensitive 25 ppb (*n* = 3) and female resistant 300 ppb (*n* = 2); Table1). Probes with a significant three-way interaction effect were removed from all other significant effect lists and clustered by *k-*means clustering based on similar expression (Euclidean distance metric; 4 clusters; Agilent). Fold changes were calculated based on marginal means for each comparison described below (i.e., controlling for other factors in the model due to unequal sample size in some conditions; Table1). Probes with a significant sex by population interaction were removed from sex or population main effect lists and fold-change-filtered (FC ≥ 1.5) between populations to identify genes specific to one sex (unchanging or discordant in other sex) or regulated to a larger extent in one sex than the other. Probes with a significant main effect of population were fold-change-filtered between resistant and sensitive populations (FC ≥ 1.5). Probes with a significant main effect of EMB concentration were fold-change-filtered against the 0 ppb control (FC ≥ 1.5).

Gene Ontology and pathway enrichment was performed in DAVID Bioinformatics using a modified Fisher's exact test (Huang et al. 2009) (*P* ≤ 0.05; number of genes in category ≥4), and result redundancy was reduced using GO Trimming (80% soft trim threshold; Jantzen et al. 2011). For this analysis, Entrez IDs assigned to contigs used for probe design (Yasuike et al. 2012) were used. Background lists for enrichment comparisons (i.e., all probes passing quality control) were specific to each experiment (Pacific = 12 085 probes; Atlantic = 15 578). Principal component analysis was performed in GeneSpring using normalized expression values for all probes passing quality control filters in Atlantic lice.

### Reverse-transcription quantitative polymerase chain reaction (RT-qPCR)

Pacific *L. salmonis* samples described above (including the 10 ppb condition) were used for RT-qPCR analysis. Each SuperScript® III (Life Technologies) reverse-transcription reaction included 2.5 μg total RNA and a 50:50 mix of random hexamers and oligo(dT)_20_ primers (total per reaction 50 ng), as per manufacturer's instructions. Resultant cDNA samples were diluted 20-fold. A standard curve for primer testing was generated from a sevenfold dilution of a sample from each condition and used in a fivefold, six-point serial dilution. All primers were tested for efficiency between 80% and 110% and for a single product by melt curve analysis and amplicon purification and sequencing as previously reported (Sutherland et al. 2011). qPCR amplification was performed using SsoFast EvaGreen with Low Rox (Bio-Rad) as per manufacturers' instructions for the MX3000P thermocycler (Agilent) using the following thermal regime: 95°C for 5 min (1 cycle); 95°C for 20 s and then 55°C for 30 s (40 cycles), followed by a melt curve (55–95°C reading fluorescence at 0.5°C increments). Samples were run in duplicate and accepted when technical replicates were within 0.5 cycles. A no template control (NTC) and -RT control was run for each gene. Although some contaminating gDNA remained after column purifications, -RT controls for each gene were more than six cycles greater than the most dilute sample for that gene and thus would have a minimal effect on quantification (Laurell et al. 2012). Genes of interest were normalized using *structural ribosomal protein s20*, a validated *L. salmonis* normalizer candidate (Frost and Nilsen 2003) in qBASEplus (Biogazelle) using primer-specific efficiencies.

Pacific salmon lice can carry a microsporidian parasite *Facilispora margolisi* at approximately 50% prevalence (Jones et al. 2012b). As the impact of the microsporidia on *L. salmonis* or on the *L. salmonis* response to EMB has not yet been characterized, to ensure that there was no confounded variation due to differences in microsporidian infection among groups, Pacific lice pools used here were tested for *F. margolisi* using diagnostic primers (Jones et al. 2012b). All pools tested positive. Additionally, a subset of samples from Atlantic *L. salmonis* was also tested, but no positives were detected, which is consistent with the observation that *F. margolisi* has not been found in Atlantic *L. salmonis* (S. Jones, *personal observation*).

Atlantic lice used in the microarray experiment were also used for RT-qPCR analysis, with the exception of one sensitive female 1000 ppb sample that did not amplify (total *n* = 76). Total RNA was reverse-transcribed using 400 ng input in iScript™ Reverse Transcription Supermix (Bio-Rad) with a mix of random hexamers and oligo(dT) primers, as per manufacturer's instructions. Subsequently, cDNA samples were diluted 10-fold. Additionally, primer efficiency was performed as described above, except that the standard curve was prepared with an initial 2-fold dilution, and a single product was confirmed for the amplicons by melt curve analysis. qPCR amplification was performed using SsoAdvanced™ SYBR® Green Supermix (Bio-Rad) as per manufacturers' instructions for the Mastercycler ep realplex thermal cycler (Eppendorf) using the following thermal regime: 95°C for 10 min (1 cycle); 95°C for 15 s, primer-specific annealing temperature (see Table S1) for 15 s, and then 72°C for 15 s (40 cycles), followed by a melt curve (55–95 °C reading fluorescence at 0.5°C increments). Samples were run in duplicate (99.63% of technical replicates within 0.5 cycles). As above, NTC did not amplify, and -RT controls were more than 6 Ct above the lowest expressed samples. Candidate normalizer genes included *structural ribosomal protein s20* (*rps20*), *elongation factor 1-alpha* (*ef1a*), and *vinculin* (*vcl*). The most stable as calculated by geNORM (Vandesompele et al. 2002) were *rps20* and *ef1a* (geNORM M-value and coefficient of variation of 0.339 and 0.118, respectively), and therefore, the geometric mean of these two genes was used to normalize genes of interest in qBASEplus (Biogazelle, Zwijnaarde, Belgium) using primer-specific efficiencies (Table S1).

For both the Pacific and Atlantic samples, the log_2_ RT-qPCR normalized value and the log_2_ microarray value for each sample were correlated in R (R Core Team 2014) using linear models with microarray values as the independent variable and RT-qPCR values as the dependent variable to obtain an adjusted R-squared value and slope of the relationship for each gene. Pacific RT-qPCR (which included the 10 ppb condition) was tested for significance by one-way anova and *post hoc* Tukey HSD in R.

## Results

### Transcriptomic effects of EMB exposure in Pacific lice

**Figure 1:**
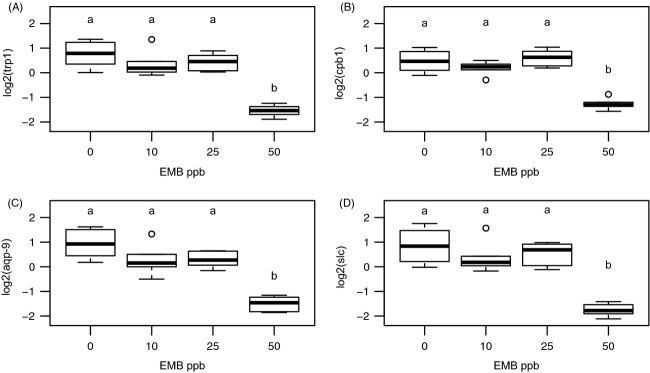
Pacific lice degradative enzyme and transporter suppression. RT-qPCR confirmed the downregulation at 50 ppb of degradative enzymes *trypsin-1* (trp-1) and *carboxypeptidase-1* (cpb-1), as well as transporters *aquaporin-9* (aqp-9) and *high-affinity copper transport protein* (slc). Conditions that do not share a letter above the boxplot are significantly different from each other (Tukey HSD *P* ≤ 0.05). Boxplot displays median and interquartile range.

The transcriptional response of Pacific lice to EMB was minimal until 50 ppb (only three differentially expressed probes at 25 ppb). Although no probes were upregulated at 50 ppb, 148 probes were downregulated (relative to either 0 or 25 ppb; Table2). All differentially expressed genes can be found in Table S2. The downregulated genes at 50 ppb (43 unique annotations) was enriched for proteolysis (11 genes; *P* = 6.7E-06) and included probes annotated as degradative enzymes including *trypsin-1*, *carboxypeptidase B*, *chymotrypsin*, *collagenase*, *acidic mammalian chitinase*, *hypodermin-B*, and others (Table3). Additional enriched functions included lipid metabolism (five genes; *P* = 0.025) and cation binding (10 genes; *P* = 0.04), among others (see Table S3); several other enzymes and transporters were downregulated. The suppression at 50 ppb EMB was confirmed by RT-qPCR, and the lack of differential expression until 50 ppb was confirmed by assessing the additional dataset at 10 ppb by RT-qPCR (Fig.1).

**Table 2:** Differential expression in Atlantic and Pacific lice responding to emamectin benzoate (EMB). Probes responding to EMB exposure in Atlantic and Pacific lice. Atlantic responses increased until 300 ppb, and Pacific lice response was minimal until 50 ppb EMB

Subspecies	Condition versus control, ppb	Probes ≥ 1.5-fold	Probes ≥ 2-fold
Atlantic	0.1	22	4
	25	236	44
	300	513	118
	1000	474	86
Pacific	25	3	3
	50	148	144

**Table 3:** Pacific lice degradative enzyme suppression from emamectin benzoate (EMB). Descriptions, corrected *P*-value, and linear fold change for genes present in Gene Ontology category proteolysis (bold font), or with known degradative function

Probe ID	Probe description	Corr. *P*-value	FC EMB 25 vs 0	FC EMB 50 vs 0
C068R042	Acidic mammalian chitinase	0.0023	–	−3.8
C036R034	**Anionic trypsin-1**	0.0035	–	−7.3
C005R080	**Anionic trypsin-2**	0.0018	–	−6.4
C102R046	**Aspartic proteinase oryzasin-1**	0.0025	–	−5.8
C020R062	Carboxypeptidase B	0.0018	–	−4.5
C009R120	Cathepsin D	0.0021	–	−5.4
C099R025	**Cathepsin K**	0.0045	–	−6.6
C022R035	Chymotrypsin BI	0.0021	–	−4.6
C123R134	Collagenase	0.0018	–	−3.8
C053R101	Gamma-glutamyl hydrolase	0.0018	–	−6.5
C133R012	**Neprilysin-2**	0.0026	–	−3.1
C100R117	**Ovochymase-1**	0.0018	–	−9.7
C158R157	**Placental protein 11**	0.0032	–	−5.0
C171R022	**Probable cysteine proteinase At3g43960**	0.0035	–	−4.0
C080R027	**Putative serine protease K12H4.7**	0.0021	–	−3.9
C028R010	**Transmembrane serine protease 8**	0.0018	–	−9.5
C042R088	Trypsin-1	0.0018	–	−2.4
C171R148	**Zinc carboxypeptidase A 1**	0.0018	–	−6.7

### EMB sensitivity differences between Atlantic lice populations

Bioassays confirmed increased resistance in the Back Bay lice relative to those from Grand Manan (Table4) and indicated the highest resistance in males. Furthermore, bioassays of the F1 generation propagated in the laboratory followed similar trends. All F1 lice were healthy-vigorous at 0, 0.1, and 25 ppb EMB. At 300 ppb, four sensitive males were healthy-vigorous, three weak (swimming but not attaching to the beaker), and one immobile (no twitching), whereas nine resistant males were healthy-vigorous and one moribund (immobile and twitching). At 300 ppb, one sensitive female was healthy-vigorous, four weak, and three immobile, whereas six resistant females were healthy-vigorous and four weak. At 1000 ppb, all lice in both populations were immobile.

**Table 4:** Survival differences between Atlantic populations. EC50 (ppb) for male and female lice from the two populations with differing emamectin benzoate sensitivity

Population	EC50 ppb (95% CI)	
	Males	Females
Back Bay (Resistant)	840 (614, 1047)	254 (218, 296)
Grand Manan (Sensitive)	63 (11, 352)	75 (13, 432)

### Comparative influence of sex, population, and EMB on Atlantic lice transcriptomes

**Figure 2:**
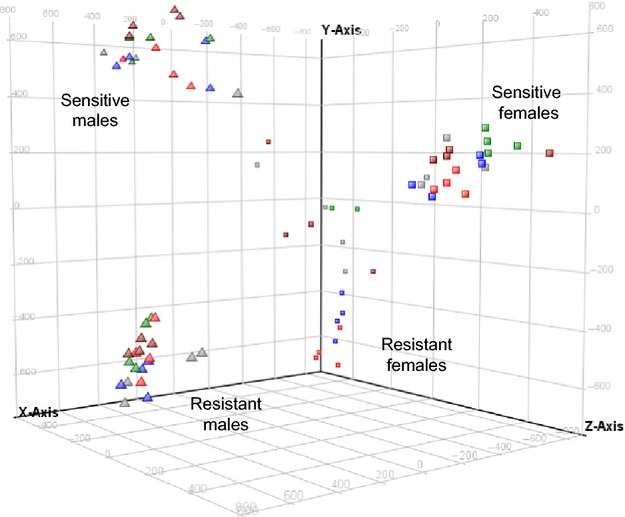
Principal component analysis of samples based on gene expression. Male and female lice samples separated along the *x*-axis (PC1: 40.9% of variation), and populations separated by y- and z-axes (PC2: 28.3% and PC3: 20.7%, respectively). A separation of emamectin benzoate (EMB) doses is identifiable in the resistant females only. Colors display EMB concentration (red = 0; blue = 0.1; gray = 25; green = 300; brown = 1000), and shape displays the sex (triangle = male; square = female); population is labeled beside each cluster.

The most influential factor affecting gene expression was sex (Fig.2; Table5; PC1: 40.9% of total variation). Population also had a large effect: Males or females clustered by population in the PCA, and many genes differed between populations (Table5). The only sex–population combination to show a large effect from EMB dose in the PCA was the resistant females (Fig.2).

**Table 5:** Overview of factors influencing Atlantic lice transcriptomes. Numbers of significant probes shown for each effect (sex, population (Pop), and emamectin benzoate concentration (EMB conc)) and interaction prior to fold change filters. Probes with a three-way interaction are not included in two-way interaction or main effect lists, and probes with a significant two-way interaction are not included in the main effect lists involved in the interaction

Comparison	Number of probes
Sex ^*^ Pop ^*^ EMB conc	151
Sex ^*^ Pop	8242
Sex ^*^ EMB conc	26
Pop ^*^ EMB conc	19
Sex	4683
Pop	3699
EMB conc	1413

The main objective of this study is to identify genes related to EMB resistance, and therefore, the most relevant genes are those (i) differing in baseline expression between populations consistently in both sexes (main effect population); (ii) differing in baseline expression between populations inconsistently in sexes (sex by population interaction); (iii) responding to EMB exposure in both sexes and populations (main effect EMB); and (iv) responding to EMB exposure specifically in one sex–population combination (three-way interaction of sex, population, and EMB). Genes in these four categories will be presented in the next four sections.

### Genes differing in baseline expression between populations in both sexes (Atlantic)

**Figure 3:**
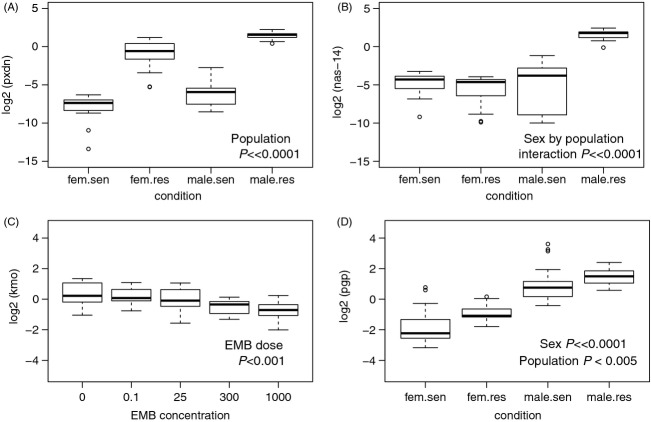
Expression of genes potentially related to resistance in Atlantic lice. (A) The expression of *peroxidasin homolog* (pxdn) was highly overexpressed in the resistant population for both sexes, and (B) *zinc metalloproteinase nas-14* (nas-14) was specifically overexpressed in resistant males. (C) In contrast to the strong differences between populations, the effect of emamectin benzoate dose was minor, although several genes were differentially expressed, including *kynurenine-3 monooxygenase* (kmo). (D) As identified in previous candidate gene approaches, here, *p-glycoprotein* (pgp) was overexpressed in the resistant population and had highest expression in males. Data in (A–C) are from the microarray and (D) RT-qPCR.

A large number of genes were differentially expressed between populations consistently in both sexes, regardless of EMB presence (main effect population; Table5). Resistant lice overexpressed 446 probes relative to sensitive (141 of which were greater than 2.5-fold). The highest overexpressed genes in resistant lice were *peroxidasin homolog* (two probes; >140-fold; Fig.3A), *collagenase*, *ovochymase-1*, *trypsin-1*, *phospholipid hydroperoxide glutathione peroxidase* (*mitochondrial*), *cathepsin D*, and *carboxypeptidase B* (>5-fold; Table S2). Overexpressed genes in resistant lice were enriched for lipid metabolic process (16 genes; *P* = 1.1E-4), response to chemical stimulus (13 genes; *P* = 0.005), catalytic activity (58 genes; *P* = 0.003), and serine-type peptidase activity (seven genes; *P* = 0.002), among others (Table S3).

Sensitive lice overexpressed 549 probes relative to resistant (134 of which were greater than 2.5-fold). The highest overexpressed probes did not have annotation (six probes > 100-fold), and the highest annotated probe was *serine protease inhibitor dipetalogastin* (10-fold). Overexpressed genes in sensitive lice were enriched for calcium ion binding (17 genes *P* = 2E-6), system development (21 genes; *P* < 0.001), cell differentiation (19 genes; *P* < 0.001), extracellular region (10 genes; *P* = 0.02), microtubule binding (five genes; *P* < 0.005), and endopeptidase activity (nine genes; *P* < 0.02), among others (Table S3). The endopeptidase activity genes included *papilin*, *stubble*, *calpain 11*, *proteasome subunit alpha type 4*, and others; this category was composed of different genes than those in the serine-type peptidase activity category that was enriched in the resistant lice overexpression list.

### Genes differing in baseline expression between populations in only one sex (Atlantic)

Resistance to EMB may be sex dependent (Igboeli et al. 2013; Jones et al. 2013; Whyte et al. 2013), and therefore, it is worthwhile to consider genes that differ between populations in only one sex (i.e., not differentially expressed or discordant in the other).

Specifically overexpressed in resistant males (but not in resistant females) were 1217 probes. Highly overexpressed were metalloproteinases *zinc metalloproteinase nas-6* and *nas-4* (FC > 50) as well as *72 kDa type IV collagenase*, *trypsin-like serine protease*, and *matrix metalloproteinase-9* (FC > 10; Table S2; Fig.3B). In comparison, 1048 probes were overexpressed in sensitive males, including many unannotated probes, or those annotated as *chitin_bind_4* and *cuticle protein cp14* (FC > 45).

Specifically overexpressed in resistant females (but not in resistant males) were 936 probes. Highly overexpressed were *tristetraproline*, *trypsin-1*, *hemicentin-1*, *trypsin-4*, and *von Willebrand factor D and EGF domain-containing protein* (FC > 50). In comparison, 913 probes were overexpressed in sensitive females. Many of these were unannotated but several with high fold change included *tyrosine aminotransferase*, *72 kda type IV collagenase*, *histone-lysine N-methyltransferase setd7*, *vitellogenin-2*, and *heparan sulfate 2-O-sulfotransferase pipe (FC > 5)*.

Additionally, there was a subset of genes differentially expressed between populations that were concordantly regulated in both sexes, but to a larger extent in one sex than the other. Male resistant lice overexpressed 146 probes to a greater extent than the female resistant lice; this list was enriched for proteolysis (Table S3) and included *chorion peroxidase heavy chain* (380-fold in males, 120-fold in females), *anionic trypsin-1*, *matrix metalloproteinase-9* (12-fold in males, 1.8-fold in females), among others (Table S2). In comparison, female resistant lice overexpressed 41 probes to a greater extent than the male lice, but this list had no functional enrichment. Male sensitive lice overexpressed 105 probes to a greater extent than female sensitive lice; this list was enriched for transition metal ion binding (Table S3) and included *a disintegrin and metalloproteinase with thrombospondin motifs 12*, *18*, and *20* (>20-fold in males, >6-fold in females), as well as *venom allergen 3* (>60-fold in males, >12-fold in females), among others (Table S2). Female sensitive lice overexpressed 73 probes to a greater extent than male sensitive lice, including *histone-lysine N-methyltransferase setd7* (25-fold in females, 3-fold in males), *nuclear pore membrane glycoprotein 210* (54-fold in females, 3-fold in males), *protein disulfide-isomerase A4* (6-fold in females, 2-fold in males), and *gamma-aminobutyric acid receptor subunit beta-like* (2.5-fold in females, 1.7-fold in males).

### Genes responding to EMB in both sexes and populations (Atlantic)

Relatively few genes responded to EMB dose. Some genes responded in both sexes and populations consistently, although most had low fold change (e.g., only 38 probes ≥ 2.5-fold at 300 ppb). Of these genes, the number of differentially expressed genes increased with EMB dose until 300 ppb; at 1000 ppb, the number and identity of differentially expressed genes were similar to that at 300 ppb (Table2; Table S2).

Among the most significantly upregulated genes from EMB exposure were *ring finger protein nhl-1*, and *alpha-* and *beta-taxilin* (FC ≥ 1.5 at 25, 300, and 1000 ppb; *P* ≤ 5E-9). However, even these genes did not have high fold change. Upregulation was also identified for genes involved in ion binding or transport, including *voltage-gated potassium channel subunit beta-2*, *sarcoplasmic calcium-binding protein* (*beta chain*), *calcium-activated potassium channel slowpoke*, *caldesmon*, and *calmodulin*.

Genes downregulated in response to EMB exposure in Atlantic lice also only had moderate fold change, including several metalloproteinases (e.g., *zinc metalloproteinase nas-4* and *nas-15*, *matrix metalloproteinase-9*, *astacin*, *72 kDa type IV collagenase*) and transporters (e.g., *solute carrier family 25 member 38*, *ABC transporter G family member 20*, and *low-affinity cationic amino acid transporter*; Table S2). Additionally, a probe annotated as *kynurenine 3-monooxygenase* was downregulated at 300 and 1000 ppb (FC > 1.5; *P* < 0.001; Fig.3C).

### Genes responding to EMB specifically in one sex–population combination (Atlantic)

**Figure 4:**
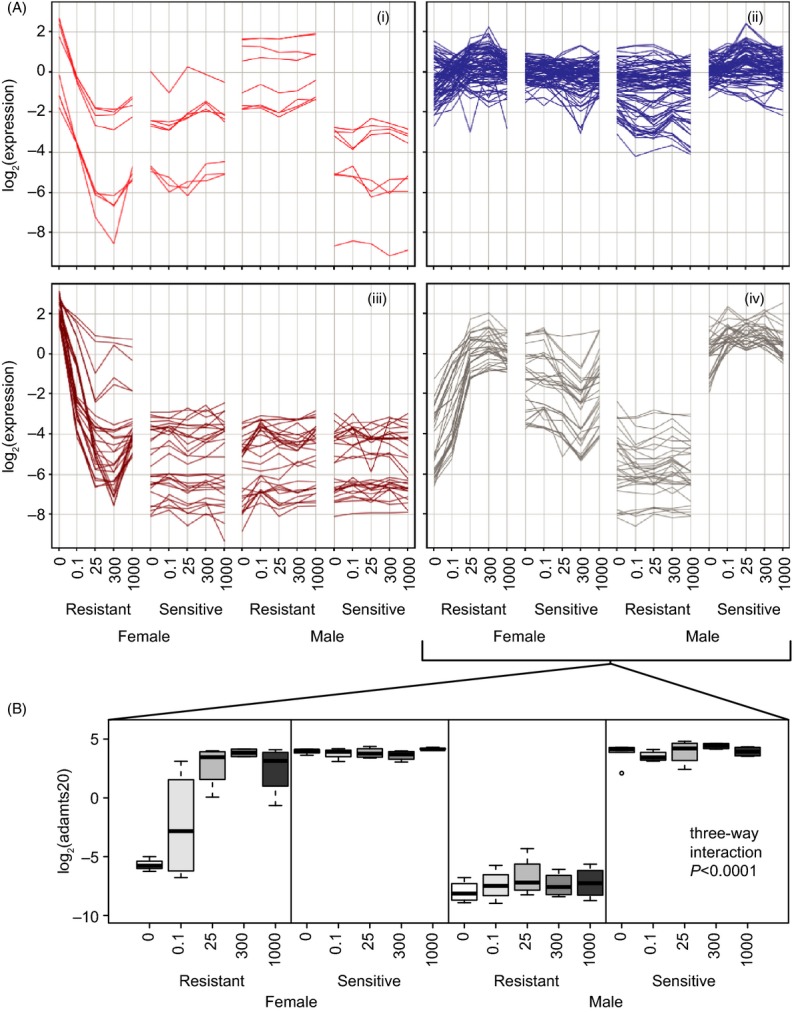
Genes responding to emamectin benzoate (EMB) specifically in resistant females. (A) Genes changing with EMB dose differently depending on sex and population were clustered based on expression into four clusters (i–iv). These genes were mainly comprised of genes responding to EMB exposure in the female resistant population. (B) The expression of a gene within cluster (iv), *a disintegrin and metalloproteinase with thrombospondin motifs 20* (adamts20) was confirmed by RT-qPCR.

A strong expression change over EMB doses was identified specifically in resistant females. These genes are likely to have contributed to the separation of resistant female samples by dose in the PCA (Fig.2). To further characterize these 151 probes, they were clustered into four clusters with similar expression (Fig.4A). Clusters (i) and (iii) contain genes with elevated expression in resistant female controls (0 ppb) and decrease either to the level of the sensitive lice or to the level of all other conditions, respectively. Cluster (iv) contains genes with low expression in resistant female controls that increases to the level of the sensitive lice. This cluster contains probes annotated as *glutenin high molecular weight subunits*, *plasmodium histidine-rich protein*, *a disintegrin and metalloproteinase with thrombospondin motifs 20*, *adhesive plaque matrix protein*, and several unknowns. Probes present in each cluster are shown in Table S2. The expression of *a disintegrin and metalloproteinase with thrombospondin motifs 20* was confirmed by RT-qPCR (Fig.4B).

### Additional RT-qPCR exploration

The expression data from the microarray were validated with RT-qPCR for the Pacific (as discussed above; Fig.1) and Atlantic lice, and most genes correlated well between the methods (Fig. S1). This included the degradative enzymes *adamts20*, *pxdn*, *nas14*, and *cpb* and transporters *slc* and *aqp-9*, as well as genes with low fold change (e.g., *hspb1*, *kcnab2*, *txlnb, kmo*; see Table S1 for full names). From these comparisons, it appeared that the RT-qPCR had a larger range than the array for the Atlantic lice (as per the slope of the relationship), whereas the opposite was true for the Pacific lice. The reason for this is not clear, but the correlation between methods was good in both subspecies. Additionally, to further test for evidence of the cellular stress response in Pacific lice, we evaluated the expression of *heat-shock protein 90* using previously characterized primers (Sutherland et al. 2012). However, consistent with the microarray analysis, no differential expression was identified at 10, 25, or 50 ppb EMB (*data not shown*).

Several interesting candidates from previous work on EMB resistance in *L. salmonis* were evaluated by RT-qPCR in the Atlantic lice, including *p-glycoprotein* (Igboeli et al. 2013), as well as *neuronal acetylcholine receptor* and *GABA-gated chloride channel* (Carmichael et al. 2013). *p-glycoprotein* was not significantly affected by EMB dose (main effect or interaction; *P* > 0.1), but had higher expression in males than females (*P* < 0.0001), and higher expression in the resistant population in both sexes (main effect population *P* < 0.005; Fig.3D), but no significant sex by population interaction effect. The expression of *GABA-gated chloride channel* was dependent on the population, sex, and dose of EMB and slightly increased over the doses of EMB in most population/sex combinations (except in male resistant lice; Fig. S2A). Over the doses of EMB *neuronal acetylcholine receptor* did not change substantially and did not have a large baseline difference between populations (Fig. S2B).

## Discussion

The regional differences in the sensitivity to EMB among populations of Atlantic *L. salmonis* (Igboeli et al. 2013) provide a system to investigate transcriptomic differences contributing to resistance mechanisms (e.g., polygenic resistance; ffrench-Constant et al. 2004). Polygenic resistance is likely in EMB-resistant *L. salmonis* (Espedal et al. 2013). The inclusion of a sensitive Pacific population in this study provides a first dataset of the induced transcriptomic response of Pacific lice to 10–50 ppb EMB, which can be indirectly compared to the Atlantic responses. However, differences between the exposures methods (e.g., pooling of multiple males and females in Pacific samples, and individual profiling in Atlantic samples) and the lice biology (e.g., presence of microsporidia infections in the Pacific lice) between the two analyses are important to consider while comparing these responses.

### Lack of induced transcriptional responses from EMB

Atlantic lice baseline expression differences were much greater in number and fold change than induced responses, in agreement with the relative contributions of baseline and induced (200 ppb EMB) transcriptomic differences in Scottish EMB-resistant and EMB-sensitive male *L. salmonis* (Carmichael et al. 2013). Interestingly, Pacific lice did not upregulate any genes in response to EMB; however, a suite of genes were downregulated in only the highest Pacific EMB dose (50 ppb). Some induced responses in resistant lice may occur; upregulation of *p-glycoprotein* occurred in response to EMB in resistant populations of *L. salmonis* (Igboeli et al. 2013). However, EMB-induced fold change may be moderate; Chilean EMB-resistant *Caligus rogercresseyi* only had moderate differences in expression of detoxification (e.g., cytochrome P450s) or transport genes (*multidrug resistance protein 1* (mrp1); Pgp1) as detected by semi-quantitative PCR (Cárcamo et al. 2011).

There was little evidence of an induced cellular stress response in the transcriptomic response to EMB in either subspecies (Atlantic or Pacific) even though many lice were exhibiting phenotypic signs of stress in bioassays at 300 and especially 1000 ppb. The lack of transcriptomic signatures of stress from the EMB exposure is a notable difference from the cellular stress response observed during salinity perturbations of copepodid *L. salmonis* (Sutherland et al. 2012). EMB-resistant Chilean *C. rogercresseyi* did not upregulate *heat-shock protein* from EMB (Cárcamo et al. 2011), and no stress response was identified in the spot prawn *Pandalus platyceros* exposed to EMB, although other biological functions were affected (e.g., transcription/translation control; Veldhoen et al. 2012). It is probable that other parasiticides with other mechanisms of action would induce different responses than those viewed here; for example, the antioxidant response of *Caligus rogercresseyi* was induced by the pyrethroid deltamethrin, which was attributed to increases in free radicals during the biodegradation of deltamethrin within the louse (Chavez-Mardones and Gallardo-Escárate 2014). Stress response genes were induced in response to the pyrethroid permethrin in pyrethroid-resistant *A. gambiae* (Vontas et al. 2005). Given the phenotypic variation that occurs even in end point detection of bioassays (Westcott et al. 2008) and in treatment response analysis (Jones et al. 2013), it will be important to continue to profile louse responses to EMB and other parasiticides, building on markers identified here, in previous work (Carmichael et al. 2013; Igboeli et al. 2013; Chavez-Mardones and Gallardo-Escárate 2014), or on markers associated with louse stress (Sutherland et al. 2012).

### Degradative enzymes and other potential resistance candidates

A main finding of the present work was the overexpression of degradative enzymes in resistant relative to sensitive lice (in both sexes), as well as the highest overexpression of many of these genes specifically in the resistant males. The highest resistance in EC50 bioassays was viewed in resistant males in the current study and in previous work (Igboeli et al. 2013), and therefore, elevated expression of degradative enzymes is associated with the most resistant condition. Interestingly, at higher doses of EMB, many of these enzymes were downregulated in both Pacific and Atlantic salmon. The resistant population may therefore be less impacted by decreases in these genes following high doses of EMB exposure. In salmon lice, secretory degradative enzymes are likely important during feeding on optimal hosts (Fast et al. 2003) and possibly during other functions.

The association of degradative enzymes with resistant phenotypes has been viewed in other species. For example, elevated baseline expression of peptidases was identified in pyrethroid-resistant *Anopheles gambiae* (Vontas et al. 2005) and in both field- and laboratory-selected DDT-resistant *Drosophila melanogaster* (Pedra et al. 2004). Functional enzymatic studies have also indicated this result, for example, with increased proteolytic activity in an insecticide-resistant house fly *Musca domestica* (Ahmed et al. 1998). Both *trypsin* and *chymotrypsin* were overexpressed in a deltamethrin-resistant population of the mosquito *Culex pipiens pallens*, and cotransfection and stable expression of these two proteins in cell culture increased cell viability in response to deltamethrin relative to controls (Gong et al. 2005). It is not clear what the function of the degradative enzymes would be in the resistance phenotype. It has been proposed that increased peptidase activity may generate energy to alleviate costs of metabolic detoxification of drugs (Ahmed et al. 1998). However, energetic costs have yet to be identified in EMB-resistant *L. salmonis* populations (Espedal et al. 2013), and costs are not always associated with resistance. For example, EMB-resistant green lacewing *Chrysoperla carnea* were reported to have more rapid development, increased fecundity, and other indicators of increased fitness relative to the sensitive control (Mansoor et al. 2013). Therefore, the reason for increased degradative enzyme expression in resistant populations requires further investigation.

Genes responding to EMB exposure in both populations may also be involved in EMB protection; it is probable that the two Atlantic lice populations used in this study are more EMB resistant than a completely sensitive strain (Igboeli et al. 2013), such as the Pacific population profiled here. Preresistant Atlantic *L. salmonis* incurred 74–100% mortality from bioassay concentrations of 30–100 ppb EMB (Tribble et al. 2007), a higher level of sensitivity than that observed in the sensitive population used here (see Table4 and Igboeli et al. 2013). Among the few genes that increased as a result of EMB exposure, two of the most significant were *alpha-* and *beta-taxilin*, which are involved in calcium-dependent exocytosis of neuroendocrine cells (Nogami et al. 2003) and potentially in promoting motor nerve regeneration (Itoh et al. 2004). As EMB acts to disrupt neurotransmission through hyperpolarization, the induction of these transcripts is noteworthy. The downregulation by EMB of *kynurenine 3-monooxygenase* is of interest because inhibition of this protein leads to an increase in the concentration of kynurenic acid and a decrease in the concentration of glutamate (Zwilling et al. 2011), the ligand for the target site of EMB (Arena et al. 1995). However, the fold changes of these transcripts were moderate, so this possible effect would have to be explored further. The identification of these genes previously unexplored in relation to EMB resistance exemplifies the potential for transcriptomics in identifying unexpected genes that may be associated with resistance (Pedra et al. 2004; Vontas et al. 2005).

In Pacific lice, the only identified response to EMB was a downregulation of genes at 50 ppb (including an enrichment for peptidase functions). Atlantic lice also decreased degradative enzymes over the concentrations of EMB, although not to the same extent. Downregulation of degradative enzymes in Pacific lice may be due to the interruption of signals for continued production of these enzymes by EMB, for example, through disruption of calcium signaling. For example, experimental calcium (Ca^2+^) influx into a rat exocrine pancreatic cell line decreased *chymotrypsin*, *amylase*, and *carboxypeptidase-a1* expression (but increased *trypsin*) (Stratowa and Rutter 1986). It is also possible that downregulation is related to EMB-induced molting (Waddy et al. 2002), as during molting suppression, it can occur for digestive enzymes *trypsin* and *chymotrypsin* (Vanwormhoudt et al. 1995; Klein et al. 1996; Sanchez-Paz et al. 2003). However, the presence of many other genes such as transporters in this list suggests this is not the case. Alternately, EMB may be acting as a antinutritional factor, which can reduce peptidase activity (e.g., in the cotton pest *Heliothis zea*; Klocke and Chan 1982), or may be reducing available energy stores leading to suppression of degradative enzymes similar to that which can occur during starvation (e.g., in white shrimp *Penaeus vannamei*; Muhlia-Almazan and Garcia-Carreno 2002). The suppression of a diverse range of transcripts further indicates interrupted signaling, but more work would need to be performed to confirm this.

Elevated *p-glycoprotein* expression in emamectin benzoate-resistant organisms has been identified in multiple species, including *L. salmonis* (Igboeli et al. 2013). Here, we also identified the highest baseline expression of *p-glycoprotein* in the most resistant condition (resistant males). However, here, we did not find evidence of *pgp* increasing over EMB doses. Our results also confirm the large influence from strain and low influence of EMB dose in Scottish resistant and sensitive *L. salmonis* (Carmichael et al. 2013). Additionally, in the Scottish populations, downregulation of degradative functions were also identified in response to EMB (e.g., hydrolase activity). In the present study, the highest fold change downregulation of degradative enzymes from EMB exposure occurred in the most sensitive population (Pacific *L. salmonis*), and in the Scottish *L. salmonis*, this was also identified in the sensitive strain (e.g., *matrix metalloproteinase-9* and *metalloproteinase*; Carmichael et al. 2013). Here, we did not find the same downregulation trends for *neuronal acetylcholine receptor* and *GABA-gated chloride channel* expression in resistant populations using the primers from the original study. However, we did identify lower baseline expression of a probe annotated as *gamma-aminobutyric acid receptor subunit beta-like* in the resistant population than the sensitive in both sexes (largest fold change in females), albeit with lower fold change than many other genes with population differences in the study. These findings may indicate differences in resistance mechanisms between Atlantic Canada and Scottish *L. salmonis* populations.

The large number of unknown genes in *L. salmonis* produces a challenge for the interpretation of *L. salmonis* transcriptomics. A potential approach to improving *L. salmonis* transcriptome interpretation may be through further characterization of co-expressed gene clusters noted here and in previous studies (Eichner et al. 2008; Carmichael et al. 2013) and using the relation to clusters and responses to environmental variables for the annotation of unknowns (Pavey et al. 2012). Continued effort in this regard may improve the interpretation of existing and future salmon lice transcriptome analysis.

### Relevance for aquaculture

The present work provides insights into the evolution of emamectin benzoate resistance in salmon lice with implications for aquaculture. Specifically, this study provides further support for the polygenic nature of emamectin benzoate resistance. This may occur incrementally among those lice exposed to a sublethal dose (ffrench-Constant et al. 2004). There are several possibilities how this may occur; for example, feeding differences may result in differential ingestion of the parasiticide by the hosts and different exposure levels to lice (Igboeli et al. 2014). The identification of markers of resistance development is an important contribution of this work. In addition to the candidates observed here, the evaluation of single nucleotide polymorphisms (SNPs) and other genomic changes associated with resistance will be an important next step.

The correlation of the resistance phenotype with elevated expression of degradative enzymes may have implications on the biology and pathology of lice. Currently, we understand that in lice, degradative enzymes are likely to function in feeding efficiency, immune evasion, and potentially in pathogenicity (Fast et al. 2003); however, it will be useful to continue to characterize the specific roles of these enzymes. The possibility that elevated expression of degradative enzymes in the resistant lice has an impact on the host–parasite interaction is an important avenue for future work.

## Conclusions

Polygenic resistance mechanisms may provide EMB protection in *L. salmonis*, and the present study identifies some potential mechanisms, most notably the association between high expression of degradative enzymes and the resistant phenotype. Induced transcriptional responses to EMB were minor and had low fold changes in comparison with baseline differences between populations differing in EMB sensitivity. Sensitive Pacific lice responded only with downregulation of enzyme and transporter gene expression. Higher doses of EMB also resulted in downregulation of degradative enzymes in Atlantic lice. Neither subspecies responded to EMB exposure with a cellular stress response. Future work on single nucleotide polymorphism (SNP) differences among populations will continue to improve our understanding of EMB resistance in *L. salmonis*, in particular the potential role for target site mutation. The interpretation of *L. salmonis* transcriptome responses (in the present study and others) may be improved with further annotation and characterization of genes and co-expressed gene clusters through meta-analysis of existing and forthcoming transcriptome studies.

## Data archiving statement

Gene expression data has been uploaded to GEO under the accession GSE56024.

## Supporting Information

**Figure S1.** RT-qPCR confirmation of microarray expression.

**Figure S2.** Exploratory RT-qPCR on previously identified targets.

**Table S1.** Primer table.

**Table S2.** Differentially expressed genes.

**Table S3.** Gene Ontology (GO) enrichment.
