# Supplementary figures and images for: Transcriptomic responses to emamectin benzoate in Pacific and Atlantic Canada salmon lice Lepeophtheirus salmonis with differing levels of drug resistance
Source: Evol Appl. 2014 Dec 22;8(2):133–48. doi: 10.1111/eva.12237 (PMC4319862; doi:10.1111/eva.12237)

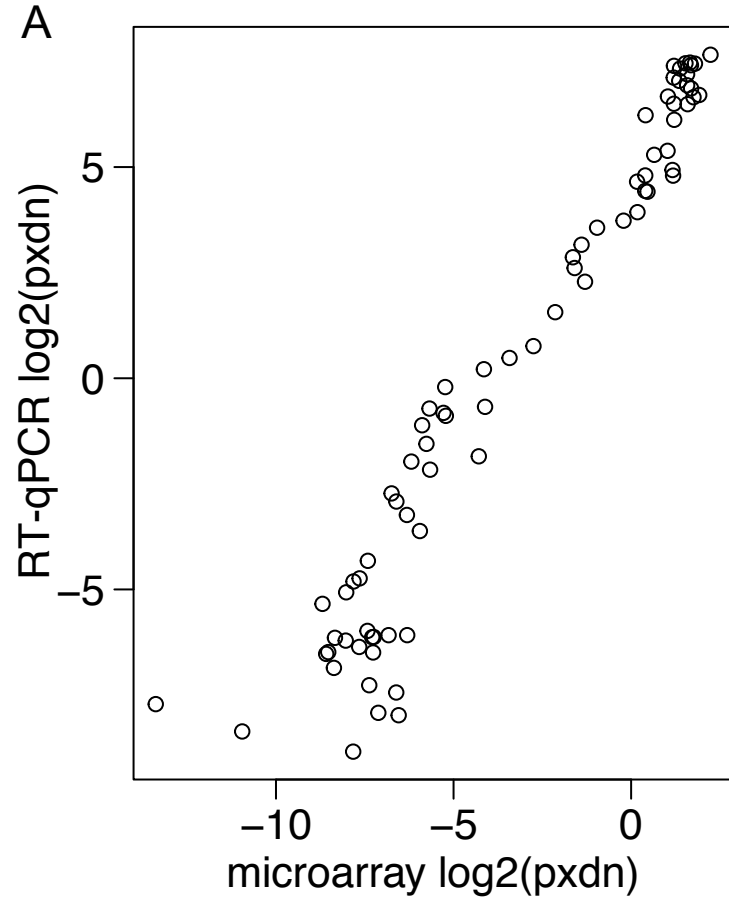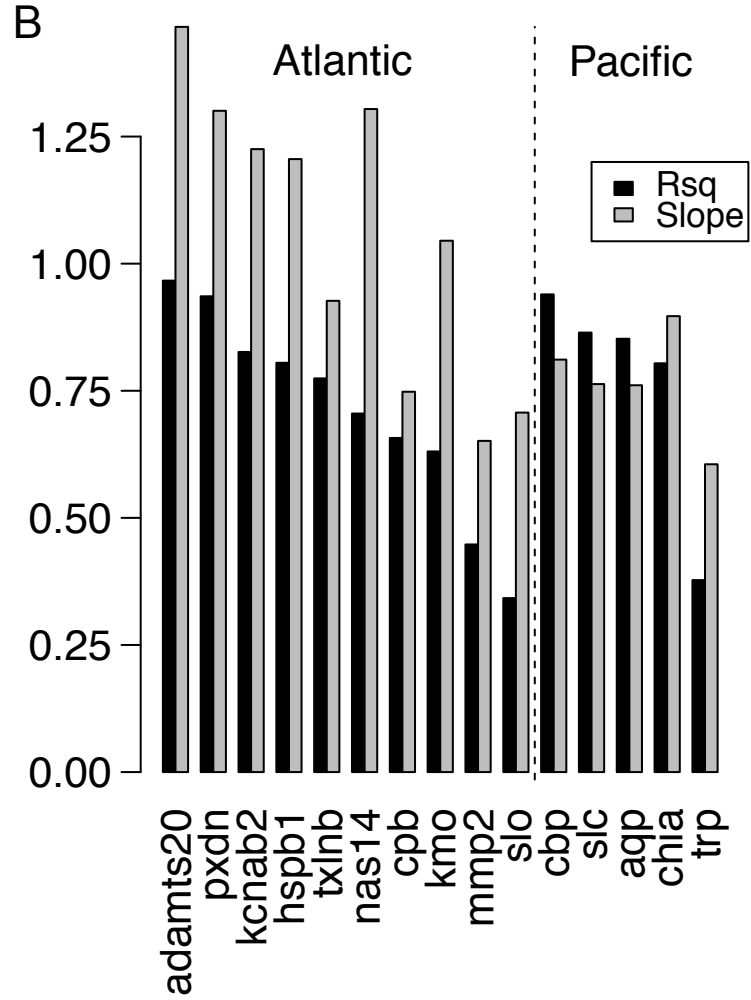

Supplement: Supplementary file 1 [file eva0008-0133-sd1.pdf]

A

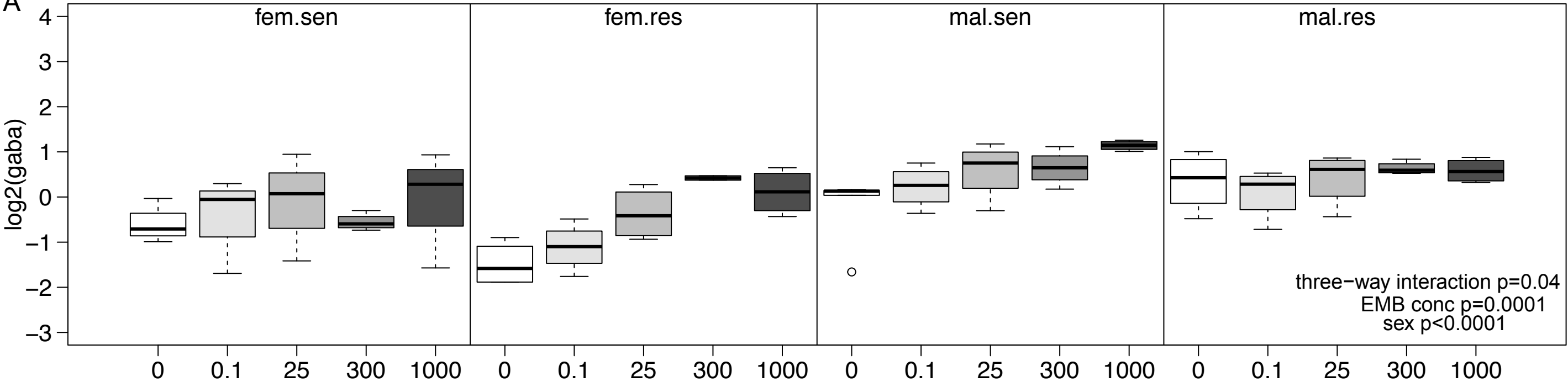

B

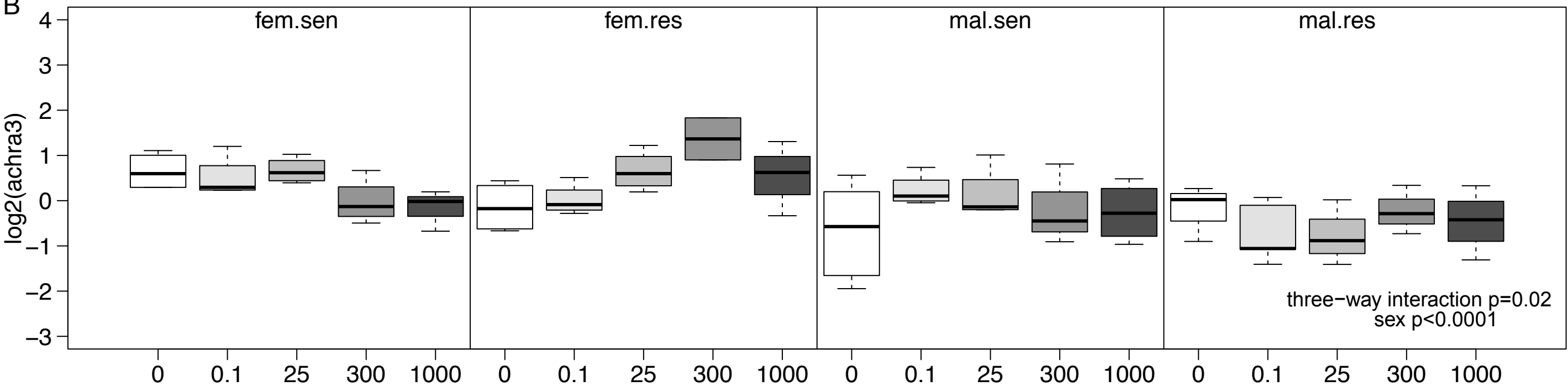

Supplement: Supplementary file 2 [file eva0008-0133-sd2.pdf]
